# Proximity to oilseed rape fields affects plant pollination and pollinator‐mediated selection on a co‐flowering plant on the Tibetan Plateau

**DOI:** 10.1111/eva.13538

**Published:** 2023-03-05

**Authors:** Yizhi Qiu, Xiaoping Yan, Hui Ma, Yuxian Wang, Rong Yang, Øystein H. Opedal, Zhigang Zhao

**Affiliations:** ^1^ State Key Laboratory of Herbage Improvement and Grassland Agro‐ecosystems, College of Ecology Lanzhou University Lanzhou China; ^2^ Northwest Institute of Eco‐Environment and Resources Chinese Academy of Sciences Lanzhou China; ^3^ Department of Biology, Biodiversity Unit Lund University Lund Sweden

**Keywords:** mass‐flowering crops, oilseed rape, plant–pollinator interaction, pollinator‐mediated selection, Tibetan Plateau

## Abstract

The ecological effects of mass‐flowering crops on pollinator abundance and species richness of neighbouring habitats are well established, yet the potential evolutionary consequences remain unclear. We studied effects of proximity to a mass‐flowering crop on the pollination of local co‐flowering plants and on patterns of natural selection on a pollination‐generalised plant on the Tibetan Plateau. We recorded pollinator visitation rates and community composition at different distances (near vs. far) to oilseed rape (*Brassica napus*) fields in two habitat types and quantified pollinator‐mediated selection on attractive traits of *Trollius ranunculoides*. The proximity to oilseed rape increased pollinator visitation in neighbouring alpine meadows and changed pollinator composition in neighbouring shrub meadows. *Trollius ranunculoides* in the alpine meadow near oilseed rape received three times more pollinator visits (mainly bees) and consequently had a 16.5% increase in seed set but also received slightly more heterospecific pollen per stigma. In contrast, pollinator visitation to *T. ranunculoides* in the shrub meadow near oilseed rape was three times lower (mainly flies), leading to a 10.7% lower seed despite no effect on pollen deposition. The proximity to the oilseed rape field intensified pollinator‐mediated selection on flower size and weakened selection on flower height of *T. ranunculoides* in the alpine meadow but did not affect phenotypic selection on either trait in the shrub meadow. Our study highlights context‐dependent variation in plant–pollinator interactions close to mass‐flowering oilseed rape, suggesting potential effects on the evolution of flower traits of native plants through altered pollinator‐mediated selection. However, context dependence may make these effects difficult to predict.

## 1 INTRODUCTION

Land‐use changes cause large effects on biodiversity and ecosystem services in natural and semi‐natural habitats (Grab et al., 2019; Newbold et al., 2015; Outhwaite et al., 2022). Biodiversity loss and other changes can be a direct result of habitat loss and fragmentation or an indirect result of changed species interactions in human‐modified landscapes. For example, the increasing cultivation of mass‐flowering crops such as oilseed rape in Europe is associated with changes in pollinator abundance and species richness (Holzschuh et al., 2016). Mass‐flowering crops provide copious and easily available flower resources and thus attract a variety of different insects (Hoyle et al., 2007), which can have direct effects on native pollinator communities and indirect effects on co‐flowering plants that rely on them (Amy et al., 2019; Carvalheiro et al., 2014). The direction of these effects is not entirely clear, however, and two contrasting patterns have been reported (Colin et al., 2019). On the one hand, pollinators in surrounding habitats can be highly attracted to the mass‐flowering crops (Grab et al., 2017; Magrach et al., 2017; Montero‐Castaño et al., 2016; Rollin et al., 2013; van Reeth et al., 2018; Woodcock et al., 2016). Competition for pollinators can thus lead to declined pollination reliability and reduced reproductive success of native plants in the surrounding landscape (Holzschuh et al., 2011; Stanley & Stout, 2014). On the other hand, pollinators may spill over from mass‐flowering crops into co‐flowering neighbours, which may increase pollinator abundance or diversity (Hagen & Kraemer, 2010; Hanley et al., 2011), and consequently improve the pollination success of neighbouring co‐flowering plants (Samnegård et al., 2011). While the ecological effects of mass‐flowering crops on co‐flowering plants have been well studied (e.g. Geslin et al., 2017; Hanley et al., 2011; Holzschuh et al., 2016), the potential evolutionary consequences remain unclear (Schroeder et al., 2021). In particular, we lack a thorough understanding of whether and how mass‐flowering crops influence the evolution of co‐flowering wild plants through altering insect communities.

Because mass‐flowering crops can affect the composition of the pollinator community in surrounding habitats (Chamberlain et al., 2013; Spiesman & Gratton, 2016), it follows that crop cultivation can influence the evolution of wild plants via altered pollinator‐mediated natural selection. Human influence on selection has been reasonably well studied (Fugere & Hendry, 2018), yet rarely in the context of agricultural landscapes (Mitchell et al., 2021). Theoretical and empirical works suggest that the strength and direction of pollinator‐mediated selection may be associated with plant–pollinator interaction strength (Bartkowska & Johnston, 2015; Benkman, 2013; Sletvold & Ågren, 2014; Vanhoenacker et al., 2013). When a plant species is highly dependent on pollinators for reproduction, pollinator‐mediated selection is critical in shaping the evolution of floral traits involved in pollinator attraction and pollen transfer (Harder & Johnson, 2009; Opedal, 2021). In agricultural landscapes, many co‐flowering wildflowers exhibit generalised pollination systems in which pollinator assemblages often vary geographically (Gómez et al., 2010; Moeller & Geber, 2005; Price et al., 2005; Zhao & Huang, 2013). Different populations of such species may be visited by pollinator species that differ in morphology, foraging behaviour and floral preference, which may result in the differentiation of floral traits among populations (Johnson & Steiner, 1997; Nilsson, 1988; Opedal, 2021). To date, very few studies have assessed how patterns of natural selection vary among plant populations within agricultural landscapes (Mitchell et al., 2021).

In recent years, a mass‐flowering landscape of oilseed rape (*Brassica napus* L.) has developed for crop production on the Tibetan Plateau, with some fields also becoming famous scenic spots during the flowering season. The ecological and evolutionary impacts of this exotic mass‐flowering crop on local pollinators and plant reproduction have not yet been investigated. At high altitudes, pollinator abundance and diversity tend to decrease, leading to intensified pollen limitation (Arroyo et al., 2006; Bingham & Orthner, 1998). Intensified pollen limitation and competition for pollinators can, in turn, generate a stronger selection of floral traits involved in attracting pollinators (e.g. Zhao & Wang, 2015). Oilseed rape attracts a wide range of insects, including bees, flies, butterflies and managed honeybees (Holzschuh et al., 2016; Kovács‐Hostyánszki et al., 2013; Stanley & Stout, 2014), which overlaps in pollination niche with most co‐flowering wild plants (Stanley & Stout, 2014). If spillover effects occur, co‐flowering wild plants adjacent to oilseed rape would receive more pollinator visits, thus alleviating pollen limitation. Alternatively, co‐flowering plants near oilseed rape might suffer intensified pollen limitation due to local pollinator decline caused by a dilution effect of oilseed rape. A long‐term effect of mass‐flowering oilseed rape on pollinator assemblages and plant–pollinator interactions in plant communities may alter pollinator‐mediated selection on flower traits of co‐flowering wild plants.

To study the ecological and evolutionary impacts of oilseed rape fields on native plants, we focused on the self‐incompatible and pollination‐generalised plant *Trollius ranunculoides*, which is a common species of grasslands adjacent to oilseed rape fields on the Tibetan Plateau. *Trollius ranunculoides* and oilseed rape overlap in the flowering period in alpine meadows in the study area (Z.G. Zhao, personal observation). Previous studies have shown that flower size variation of *T. ranunculoides* aligns with the variation in visitation rates by bees and flies, and that flower traits were subject to intensified selection under lower pollinator availability (Zhao & Huang, 2013; Zhao & Wang, 2015). Moreover, the flower size of *T. ranunculoides* varied among different habitats, which is likely associated with multiple factors (Zhao et al., 2007). Here, we specifically studied pollinator‐mediated selection in *T. ranunculoides* populations with different distances (near vs. far) to oilseed rape fields. To assess generality, we replicated the study in two locally common habitat types, alpine meadow and shrub meadow. We asked (1) whether pollinator abundance and composition differ between habitats near vs. farther from oilseed rape fields; (2) whether *T. ranunculoides* individuals adjacent to oilseed rape receive more conspecific pollen and have higher seed set, as expected if oilseed rape has a spillover effect of increasing local insect diversity and abundance and (3) whether patterns of the net and pollinator‐mediated selection on attractive traits (flower height and flower size) differ between *T. ranunculoides* populations near vs. farther from oilseed rape fields.

## 2 MATERIALS AND METHODS

### 2.1 Study species and study site

*Trollius ranunculoides* Hemsl. is a hermaphroditic perennial herb, widely distributed in alpine areas of the Tibetan Plateau. Plants generally bloom from June to July and a single flower lasts 8 days on average. Each plant generally produces a single bright yellow flower on an erect stalk 6–20 cm high, yet a few individuals produce two or three flowering stalks. The bowl‐shaped flower comprises five yellow petal‐shaped sepals and several stamen‐shaped golden yellow petals. *Trollius ranunculoides* is self‐incompatible, and seed production depends on insect pollinators including bees, flies, ants and occasionally beetles (Zhao et al., 2007).

The present study was conducted in Zhuoni County in the northeast of the Tibetan Plateau. The mean annual temperature is 2°C, and the mean annual precipitation is 550 mm. Oilseed rape has been cultivated in the surrounding countryside since 2011. We chose seven alpine meadow sites and three shrub meadow sites (dominated by *Potentilla fruticosa*) adjacent to oilseed rape fields (Table S1).

### 2.2 Pollinator surveys

We studied pollination ecology and pollinator‐mediated selection on the flower traits of *T. ranunculoides* from June to September 2019. To assess whether pollinator abundance and community composition differ between habitats near vs. farther from oilseed rape fields, we selected paired study plots at each site (10 pairs in total) so that each pair had one plot adjacent to the oilseed rape field (<100 m), and the other plot farther from oilseed rape (>700 m). Each plot pair had a homogeneous community composition and a consistent local environment. In each plot, we randomly chose three transects (50 m × 2 m and 15 m apart) for pollinator surveys. We recorded, using a camera, pollinators visiting flowering plants during a 30 min walk along each transect. A successful visit was defined as the insect body contacting reproductive organs (i.e. anthers or stigmas) of flowers. Field observations were made between 10:00 am and 17:00 pm on sunny days. All insects and the visited plants were identified in the photos. We divided the insects into five groups: Diptera, Hymenoptera, Lepidoptera, Coleoptera and others (e.g. locusts, spiders). We calculated the community‐level pollinator abundance as the number of visits per hour in each transect (visits/h), and the visitation rate to *T. ranunculoides* as the number of pollinator visits divided by the number of open flowers counted in the 100‐m^2^ transect (visits/h/flower).

### 2.3 Selection study

To study pollinator‐mediated selection on the flower traits of *T. ranunculoides*, we manipulated the pollination regime (open pollination vs. supplemental hand pollination) at different distances from the oilseed rape field (near vs. far) in a factorial design. We replicated the experiment at one alpine meadow site and one shrub meadow site (Table S1). Both sites are located close to the same large oilseed rape field (5 ha) that has been continuously planted for more than 8 years. At each site, we studied one *T. ranunculoides* population near the oilseed rape field, and another farther from the oilseed rape field. Plant community composition and flower density were similar between the ‘near’ and ‘far’ populations. In June 2019, we randomly chose 150–200 plants with flower buds in each population and assigned them to one of two experimental pollination treatments. Individuals assigned to the open‐pollination (control) treatment were left unmanipulated. Individuals assigned to the supplemental hand‐pollination treatment were hand‐pollinated as they became receptive by brushing each stigma with dehiscing anthers from multiple donor plants and saturating the stigma surface with pollen. We collected pollen from donor plants outside the experimental plots. All hand‐pollinated flowers received supplemental pollen at least twice to ensure successful pollination (Chapurlat et al., 2015), and may also have received natural pollination via insects.

For each plant, we measured flower height (distance from the ground to the flower) to the nearest millimetre and corolla diameter (i.e. flower size) of a fully open flower to the nearest 0.1 mm with digital callipers. Additionally, we randomly selected 15–20 naturally pollinated flowers of *T. ranunculoides* in each population. We stained the stigmas with magenta gel and counted the number and type of pollen on the stigmas under the microscope. When the fruits matured, we collected the fruits of all marked flowers to count the number of seeds and unfertilised ovules. All seeds were observed under a stereoscope to record the number of mature seeds, sterile seeds and unfertilised ovules. We calculated the proportional seed set as the ratio of the number of mature seeds to the number of total ovules per fruit. We quantified female reproductive fitness as the number of mature seeds per individual.

### 2.4 Statistical analyses

We used ANOVA to assess differences in visitation rate between plots near vs. far from oilseed rape fields, and non‐metric multidimensional scaling (NMDS) based on Bray‐Curtis distances to analyse differences in pollinator community composition. We used two‐way ANOVA to examine the effects of pollination treatment (C, open‐pollinated control vs. HP, supplemental hand pollination), distance to oilseed rape field (near vs. far), and their interaction on the seed set of *T. ranunculoides*. In this model, a statistically significant pollination treatment × distance interaction demonstrates that the degree of pollen limitation differs between sites located near vs. far from oilseed rape fields.

To estimate phenotypic selection gradients following the approach of Lande and Arnold (1983), we fitted multiple‐regression models with relative female fitness (seed number per plant/population mean seed number) as the response variable and standardised trait values as explanatory variables. We standardised the traits (flower height and flower size) to a mean of 0 and a variance of 1, and thus report variance‐standardised selection gradients. We estimate directional selection gradients *β*_i_ from multiple‐regression models including only linear terms, separately for each population. We quantified pollinator‐mediated selection by subtracting the estimated selection gradient for plants receiving supplemental hand pollination (*β*_HP_) from the estimate obtained for open‐pollinated controls (*β*_C_) for each trait, Δ*β*_poll_ = *β*_C_ – *β*_HP_ (Sletvold, 2019; Sletvold et al., 2010). We obtained standard errors for Δ*β*_poll_ as ${\mathrm{SE}}_{\Delta \beta \text{poll}}$ = $\sqrt{{\left({\mathrm{SE}}_{\beta_{C}}\right)}^{2}+{\left({\mathrm{SE}}_{\beta_{\mathrm{HP}}}\right)}^{2}}$. To assess whether the distance to oilseed rape and the pollination treatment influenced linear selection gradients, we fitted an ANCOVA model with relative female fitness as the dependent variable and the standardised traits (flower height and flower size), distance to the oilseed rape (near vs. far), pollination treatment (C vs. HP) and trait × distance, trait × pollination treatment and trait × distance × pollination treatment interactions as independent variables. In this model, a statistically significant trait × pollination treatment interaction indicates support for pollinator‐mediated selection, while a significant three‐way interaction term demonstrates that the strength of pollinator‐mediated selection depends on proximity to oilseed rape. We assessed potential multicollinearity through variance inflation factors (VIF), which were all < 10 indicating no serious multicollinearity.

All statistical analyses were conducted with R version 4.2.1 (R Core Team, 2022). Diagrams were drawn using GraphPad Prism 9.

## 3 RESULTS

### 3.1 Changes in pollinator abundance and assemblage composition in habitats adjacent to oilseed rape fields

**FIGURE 1:**
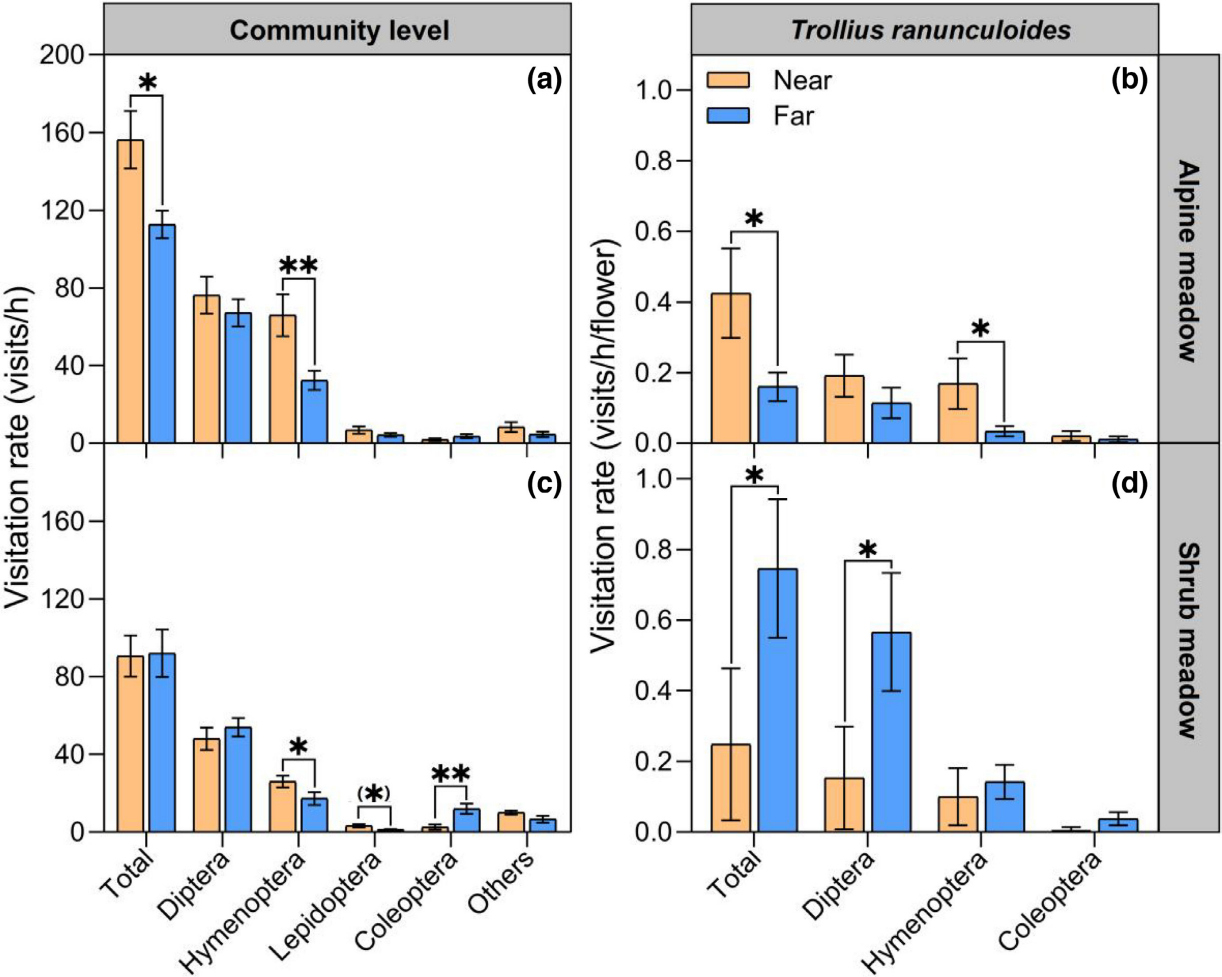
Pollinator abundance as indicated by visitation rate of all insects (‘total’) and main functional groups to flowering plants in alpine meadows (a) and shrub meadow (b) and to *Trollius ranunculoides* in alpine meadow (c) and shrub meadow (d) respectively, near vs. far from oilseed rape. Bar plots show mean ± SE. Symbols above individual bars indicate the statistical difference between near and far treatments. *p < 0.05, **p < 0.01, ***p < 0.001, (*), 0.05 < *p* < 0.1.

The pollinator abundance and assemblages of natural habitats near oilseed rape fields were different from those of habitats farther from oilseed rape fields, but the patterns differed between the two habitat types. In the alpine meadow habitat, the total pollinator activity was 38.7% higher at sites close to oilseed rape fields than at sites far from oilseed rape fields (156.29 ± 6.93 visits/h vs. 112.67 ± 3.44 visits/h, *p* = 0.035, Figure 1a). This difference arose because of two times greater abundance of Hymenoptera at sites close to oilseed rape fields (near vs. far, 66.00 ± 10.80 vs. 32.38 ± 4.94, *p* = 0.005), while we failed to detect a difference in pollinator community composition (Figure S1a). The same pattern held for *T. ranunculoides*, with a nearly three‐fold increase in the visitation rate at sites near oilseed rape fields (0.43 ± 0.13 vs. 0.15 ± 0.04), especially for Hymenoptera (Figure 1b).

In the shrub meadow habitat, the total pollinator activity was similar at sites near vs. far from oilseed rape fields (90.00 ± 5.29 vs. 91.33 ± 6.11, *p* = 0.687) (Figure 1c). Several taxa were either more (Hymenoptera, Lepidoptera) or less (Coleoptera) frequent visitors at sites close to oilseed rape fields, however, resulting in a detectable difference in pollinator assemblage composition (Figure S1b). For *T. ranunculoides*, visitation rates were three times lower (0.25 ± 0.10 vs. 0.75 ± 0.20) at sites near oilseed rape fields, mostly due to less frequent visits by flies (Figure 1d).

### 3.2 Pollen deposition and seed set of *T. ranunculoides*

The stigma pollen loads of *T. ranunculoides* were not detectably different at sites near vs. far from oilseed rape fields in either habitat type, but we recorded more heterospecific pollen (including pollen of oilseed rape) and consequently a slightly decreased proportion of conspecific pollen per stigma at alpine meadow sites near oilseed rape fields (Table 1).

**TABLE 1:** Pollen number per stigma (mean ± SE) of *Trollius ranunculoides* in two habitats with different distances (near vs. far) to oilseed rape.

	Alpine meadow			Shrub meadow		
	Near (*N* = 16)	Far (*N* = 20)	*p*	Near (*N* = 13)	Far (*N* = 20)	*p*
Total	156.48 ± 24.96	139.98 ± 17.23	0.567	135.08 ± 18.44	194.75 ± 23.16	0.131
Conspecific pollen	151.89 ± 24.54	138.64 ± 17.14	0.610	133.62 ± 18.29	193.40 ± 23.06	0.131
Heterospecific pollen	**4.59 ± 0.67**	**1.34 ± 0.31**	**<0.001**	1.54 ± 0.31	1.50 ± 0.29	0.450
Pollen of OSR	1.00 ± 0.19	0.82 ± 0.28	0.147	0.31 ± 0.21	0.25 ± 0.12	0.162
PCP	**0.96 ± 0.01**	**0.99 ± 0.002**	**<0.001**	0.99 ± 0.003	0.99 ± 0.002	0.161

*Note*: The variable PCP is the proportion of conspecific pollen. Bold indicates statistically significant effects (*p* < 0.05).

**FIGURE 2:**
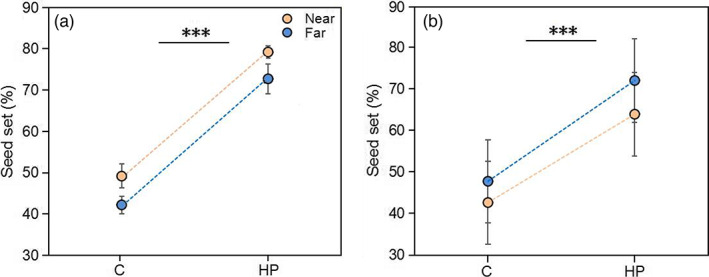
Effects of the distance to oilseed rape field (near vs. far) and the hand‐pollination treatment (open‐pollinated control [C] vs. supplemental hand pollination [HP]) on seed set (mean ± SE) of *Trollius ranunculoides* in alpine meadows (a) and shrub meadow (b). Symbols above the short bars indicate the statistical difference between C and HP. ***p < 0.001.

The mean seed set of *T. ranunculoides* differed between sites near vs. far from oilseed rape fields, but in opposite directions in the two habitat types (Table S2; Figure 2). The *T. ranunculoides* population near oilseed rape had a 16.5% higher seed set than the population far from oilseed rape at the alpine meadow site, but a 10.7% lower seed set at the shrub meadow site (Figure 2a). These differences were independent of the supplemental pollination treatment, which detectably increased seed set in both habitats regardless of the distance to the oilseed rape field (Figure 2; Table S2). Hence, the degree of pollen limitation of *T. ranunculoides* individuals did not differ between populations near and far from oilseed rape fields.

### 3.3 Pollinator‐mediated selection on the flower traits of *T. ranunculoides*

**FIGURE 3:**
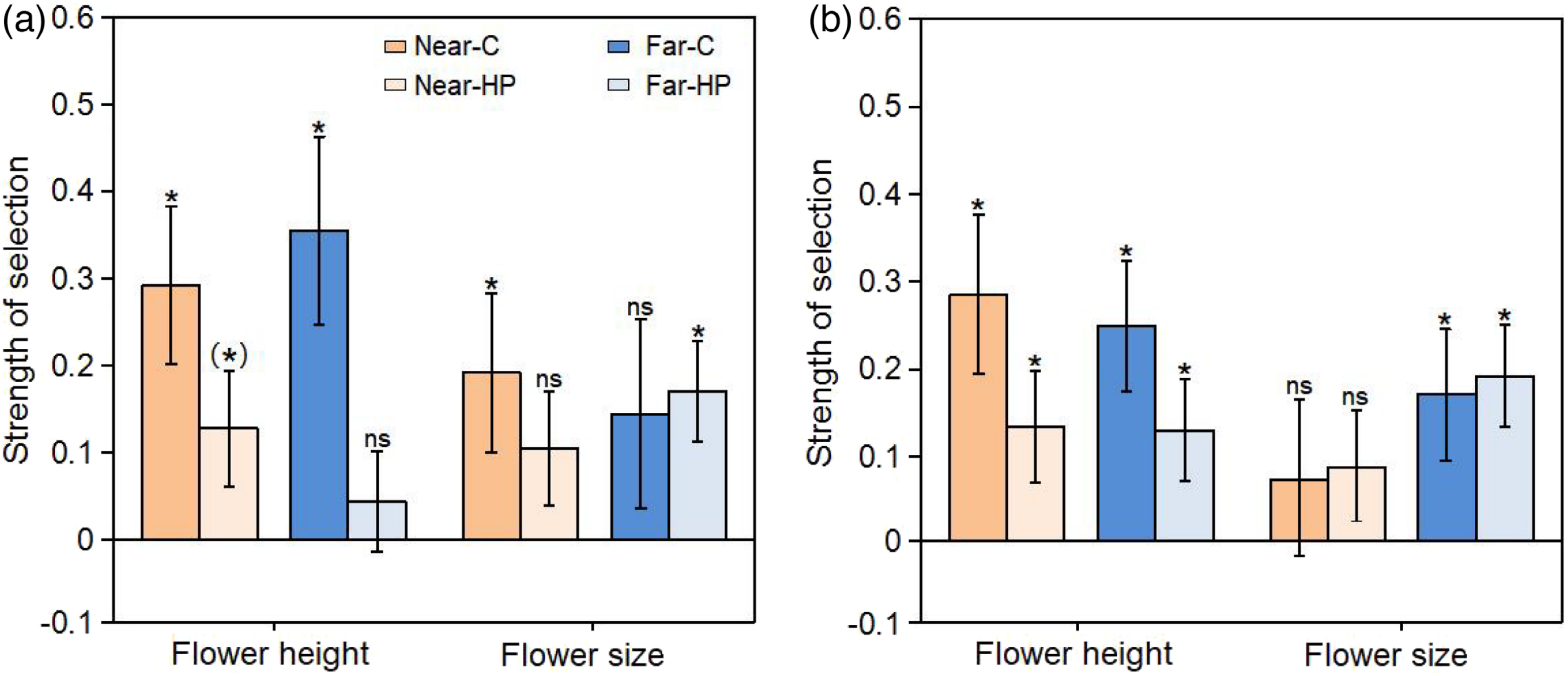
Variance‐standardised linear phenotypic selection gradients for flower height and flower size in open‐pollinated control plants (C) and plants receiving supplemental hand pollination (HP) in the *Trollius ranunculoides* populations in alpine meadows (a) and shrub meadow (b) with different distances from oilseed rape (near vs. far). Symbols above individual bars indicate statistical significance. *p < 0.05, **p < 0.01, ***p < 0.001, (*), 0.05 < *p* < 0.1; ns, not statistically significant.

**FIGURE 4:**
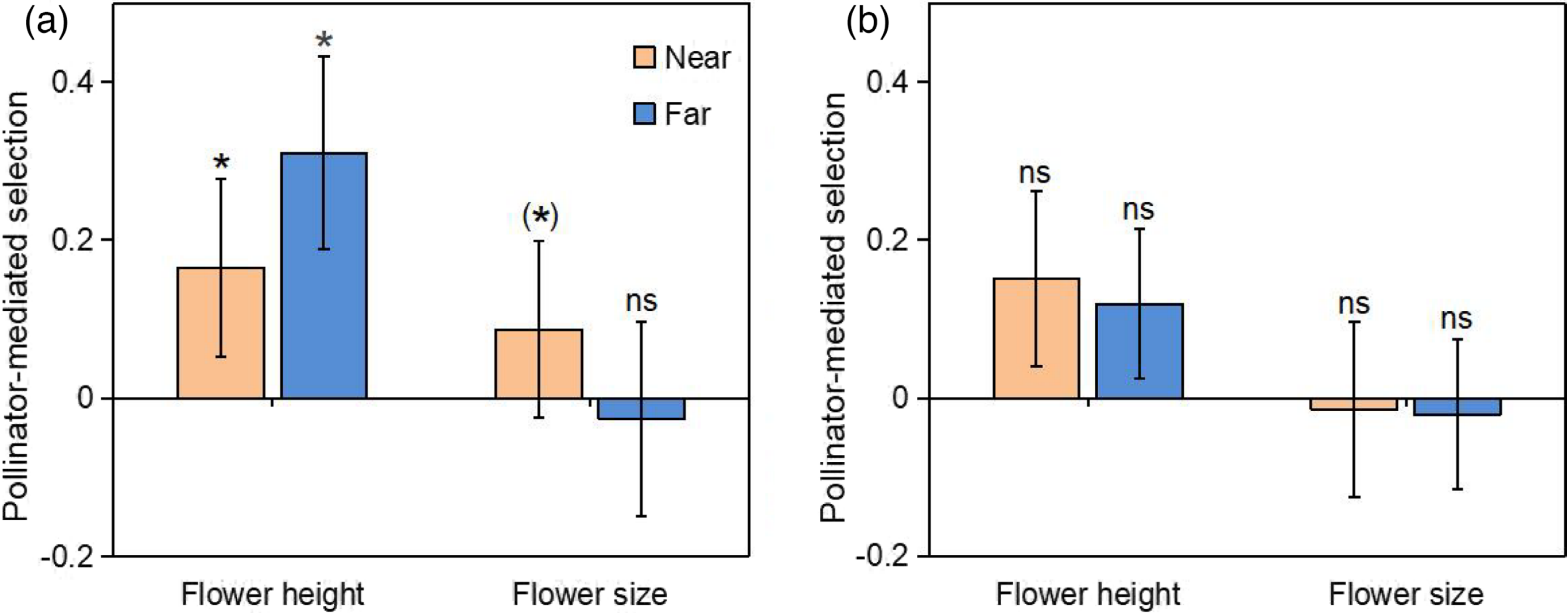
The strength of pollinator‐mediated selection (Δ*β*_poll_) on flower height and flower size in the *Trollius ranunculoides* populations in alpine meadows (a) and shrub meadow (b) with different distances from oilseed rape (near vs. far). Symbols above individual bars indicate statistical significance. *p < 0.05, **p < 0.01, ***p < 0.001, (*), 0.05 < *p* < 0.1; ns, not statistically significant.

We detected directional selection for greater flower height in the *T. ranunculoides* populations in both habitats (Figure 3; Table S3), and the strength of selection was similar at sites near and far from oilseed rape fields (non‐significant trait × distance interaction, both *p* > 0.60, Table S4). Pollinators contributed detectably to the selection on flower height in the alpine meadow (Table S3), accounting for 56.5% of the net selection in the population near oilseed rape, and 87.9% in the population farther from oilseed rape (Figure 4a, Table S3). A similar tendency for positive pollinator‐mediated selection occurred in both populations in the shrub meadow, though with weaker statistical support (Figure 4b, Table S3).

We detected directional selection for larger flower sizes in both habitats, yet the patterns differed. In the alpine meadow, pollinators accounted for 45.6% of the selection in the population near oilseed rape (Figures 3a, 4a, Table S3). In the population far from oilseed rape (Figure 4a), and in both shrub meadow populations, we did not detect pollinator‐mediated selection (Figures 3b, 4b, Table S3). We detected no significant trait × distance, pollination × distance, or trait × pollination × distance interactions in either habitat (Table S4).

## 4 DISCUSSION

### 4.1 Variation in pollinator assemblages and plant reproduction near oilseed rape fields

Pollinator visitation rates, assemblage composition, and plant reproduction differed between sites near vs. far from oilseed rape fields. However, the patterns of variation differed depending on the habitat type. In alpine meadows, we recorded a higher visitation rate of pollinators, especially Hymenoptera, in plots closer to oilseed rape fields. Particularly, *T. ranunculoides* populations near oilseed rape received three times more pollinator visits, mainly by bees. Correspondingly, the seed set of *T. ranunculoides* individuals in alpine meadows was 16.5% greater when close to oilseed rape, although the proportion of conspecific pollen declined slightly at these sites due to more heterospecific pollen deposition onto stigmas. In shrub meadows, proximity to the oilseed rape field did not change the total pollinator visitation. Instead the pollinator assemblage composition varied, with increased rates of visitation by Hymenoptera and Lepidoptera, and reduced rates of visitation by Coleoptera. By contrast, pollinator visitation to *T. ranunculoides* near oilseed rape was three times less (mainly flies), which resulted in a 10.7% lower seed set of *T. ranunculoides* individuals.

Previous studies have reported a ‘spillover effect’ of mass‐flowering crops like oilseed rape, that is higher pollinator abundance in neighbouring habitats (Blitzer et al., 2012; Geslin et al., 2017; Hanley et al., 2011), often accompanied by an increase in the fruit or seed set of plants in these habitats (Cussans et al., 2010; Kovács‐Hostyánszki et al., 2013). The opposite effect of oilseed rape has also been reported, that is pollinator dilution, in which declined pollinator abundance in the surrounding landscape may lead to competition with native plants for generalist pollinators and reduced reproductive success of co‐flowering species in natural habitats (Holzschuh et al., 2011; Stanley & Stout, 2014). Our results are partly consistent with both these patterns and further suggest that the effect of oilseed rape depends on the composition of the co‐flowering community. *Trollius ranunculoides* at alpine meadow sites near oilseed rape had higher bee visitation and seed set, indicating a spillover effect. In contrast, reduced fly visitation to *T. ranunculoides* in shrub meadow sites near oilseed rape indicated a pollinator dilution effect. The latter might be an indirect effect because total visitation and fly visitation did not change at the sites near oilseed rape. This suggests that changed pollinator community composition in shrub meadows close to oilseed rape may lead to a distortion in plant–pollinator interactions and shift flies attracted to *T. ranunculoides* to other food plants. In fact, we found, in the shrub meadow near oilseed rape, that fly visitation to *Potentilla fruticosa* and *Anaphalis lactea* increased 64% and 100%, respectively (*P. fruticosa*, near vs. far, 0.629 vs. 0.384 visits/h; *A. lactea*, 2.00 vs. 1.00 visits/h, unpublished data). Therefore, further studies are needed to examine the effects of oilseed rape on plant–pollinator interaction networks and the ecological consequences for the reproductive success of plant communities in different habitats. Previous work has also suggested that the effects of oilseed rape on pollinator abundances and pollination success in neighbouring habitats might depend on landscape‐level aggregation of oilseed rape (van Reeth et al., 2019), the flowering time of crops and wild plants and the wild plant species (Hanley et al., 2011; Kovács‐Hostyánszki et al., 2013). Together, these observations suggest that the effect of crops on co‐flowering plants may be context dependent and thus difficult to predict. Finally, *T. ranunculoides* populations were pollen‐limited across all study sites, irrespective of the distance to oilseed rape. The supplemental pollination increased the seed set from 42–49% to 63–79%, suggesting potential for pollinator‐mediated selection, but also that female reproductive success of *T. ranunculoides* individuals may be limited by resource availability other than pollen (Ashman et al., 2004; Haig & Westoby, 1988).

### 4.2 Variation in pollinator‐mediated selection

We detected both net and pollinator‐mediated selection on both advertisement traits (flower height, flower size) of *T. ranunculoides*, and the patterns of selection differed somewhat across habitats near vs. far from the oilseed rape field. Especially, pollinator‐mediated selection on attractive traits tended to be stronger in the alpine meadow than in the shrub meadow. Furthermore, pollinator‐mediated selection for flower size tended to be stronger in the population close to the oilseed rape field (near vs. far, 0.087 vs. −0.026), while selection on flower height tended to be stronger in the population far from oilseed rape (near vs. far, 0.165 vs. 0.311). The effect of mass‐flowering crops on pollinator abundance and composition in neighbouring habitats detected both in our study and in previous work (e.g. Holzschuh et al., 2016), suggests indirect effects on patterns of selection. In generalised pollination systems, variation in pollinator assemblages could change selection on advertisement traits due to pollinator preferences (Gómez et al., 2008) and thus pollinator‐mediated selection (Albertsen et al., 2021; Opedal, 2021; Zhao & Wang, 2015). Therefore, mass‐flowering oilseed rape may influence the evolution of wild species via altered natural selection mediated by pollinators arising from changed plant–pollinator interactions. One study examining how the presence of crop sunflowers (*Helianthus annuus*) alters natural selection on reproductive traits of wild sunflowers (*Helianthus annuus texanus*) reported limited evidence that proximity to crop sunflowers altered selection on flower traits (appearing for only three of eleven traits) (Mitchell et al., 2021). The effect of mass‐flowering crops also resembles exotic plants to some extent. For example, Beans and Roach (2015) showed that the presence of invasive jewelweed altered phenotypic selection on corolla height in the native *Impatiens capensis*. In the present study, *T. ranunculoides* populations at the alpine meadow near oilseed rape were subject to pollinator‐mediated selection on flower size, which may have resulted from an increased abundance of bees. Our previous study indicated a more intense selection on flower diameter in a *T. ranunculoides* population in a similar habitat, where bees preferring large flowers are more abundant (Zhao & Huang, 2013). By contrast, selection on flower height was consistently stronger and the proportion that was pollinator‐mediated was higher in the *T. ranunculoides* population at the alpine meadow far from oilseed rape, which may be caused by lower pollinator visitation and stronger pollen limitation. Flower height, as an important advertisement, strongly influence pollination success and is often subject to pollinator‐mediated selection in entomophilous plants (O'Connell & Johnston, 1998; Opedal, 2021; Sletvold et al., 2010, 2013).

An important insight from our work is that changes in pollinator assemblages and selection with distance from oilseed rape fields can depend on habitat type. In contrast to the patterns observed in the alpine meadow, proximity to oilseed rape did not detectably alter the selection on flower height and flower size of *T. ranunculoides* in the shrub meadow. In this habitat, both traits were subject to non‐pollinator‐mediated selection, as directional selection gradients for flower height and flower size did not disappear with supplemental pollination. Although *T. ranunculoides* individuals were still pollen‐limited in the shrub habitat, selection by other agents rather than pollinators may be more important (Sletvold, 2019). This difference between habitats in pollinator‐mediated selection might be linked to ecological context. It has been shown that spatial variation in selection is likely associated with environmental factors, for example vegetation height (Sletvold et al., 2013), temperature (Totland, 2001) and soil microenvironment (Caruso et al., 2003). Because seed production of *T. ranunculoides* might be simultaneously limited by both the availability of pollen and the abiotic resources, net selection may depend on the relative importance of these factors in a specific situation (Sletvold et al., 2017). Thus, the observed selection in the shrub meadow may represent resource‐ rather than pollinator‐mediated selection on flower height and flower size.

## 5 CONCLUSIONS

This study has shown that pollination environments, plant reproduction and patterns of selection can differ between different habitat types near vs. far from oilseed rape fields. In alpine meadows, increased bee visitation and seed set of the pollination‐generalised native plant *T. ranunculoides* suggests a spillover effect of oilseed rape, although slightly more heterospecific pollen arrived on stigmas. In shrub meadows, however, the reduced seed set of *T. ranunculoides* suggests a dilution effect of oilseed rape, possibly by indirectly affecting fly visitation. Correspondingly, the strength of pollinator‐mediated selection on attractive traits of *T. ranunculoides* also varied between habitat types. *Trollius ranunculoides* in the alpine meadow adjacent to the oilseed rape field was subject to stronger pollinator‐mediated selection for flower size, while selection on flower traits of *T. ranunculoides* in the shrub meadow did not change between populations near vs. far from oilseed rape. Therefore, wild plants can experience differential selection following human‐mediated land‐use change. Our study highlights context‐dependent variation in plant–pollinator interactions close to the mass‐flowering oilseed rape, suggesting a potential effect on the evolution of flower traits of native plants through altering pollinator‐mediated selection.

## FUNDING INFORMATION

National Key Research and Development Program of China (grant/award number: 2017YF050400); National Natural Science Foundation of China (grant/award numbers: 31570229 and 31870411).

## CONFLICT OF INTEREST STATEMENT

The authors declare that they have no conflict of interest.

6

## Supporting information


Data S1:

## Data Availability

All data for this study are provided in Dryad linked here: https://doi.org/10.5061/dryad.7pvmcvdz8.
