# Supplementary material for: Proximity to oilseed rape fields affects plant pollination and pollinator‐mediated selection on a co‐flowering plant on the Tibetan Plateau
Source: Evol Appl. 2023 Mar 5;16(4):814–23. doi: 10.1111/eva.13538 (PMC10130553; doi:10.1111/eva.13538)
Supplement: Supplementary file 1 — Data S1: [file EVA-16-814-s001.docx]

**Table S1** Location and characteristics of the study sites. Asterisks (*) indicate the focal sites chosen for the phenotypic-selection study.

| Habitat | Site | Latitude  (N) | Longitude  (E) | Altitude  (m) | Dominant species |
| --- | --- | --- | --- | --- | --- |
| Alpine meadow | *1 | 34°50´11´´ | 103°10´09´´ | 3092 | *Carex capillifolia*, *Lancea tibetica*, *Medicago ruthenica* |
|  | 2 | 34°50´15´´ | 103°10´30´´ | 3132 | *Carex capillifolia*, *Argentina anserina*, *Descurainia sophia* |
|  | 3 | 34°49´05´´ | 103°09´45´´ | 3196 | Poa pratensis, *Carex capillifolia,* Geranium pylzowianum |
|  | 4 | 35°00´01´´ | 103°04´56´´ | 2996 | Poa pratensis, Medicago ruthenica, *Carex capillifolia* |
|  | 5 | 34°51´36´´ | 103°05´30´´ | 3136 | *Carex capillifolia*, Poa pratensis, *Lancea tibetica* |
|  | 6 | 34°59´54´´ | 103°02´21´´ | 3177 | *Carex capillifolia*, *Argentina anserina*, *Leontopodium dedekensii* |
|  | 7 | 35°00´06´´ | 102°58´02´´ | 3062 | Poa pratensis, *Carex capillifolia*, *Medicago ruthenica* |
| Shrub meadow | *1 | 34°50´13´´ | 103°10´09´´ | 3110 | Potentilla fruticosa, Poa pratensis, *Carex capillifolia* |
|  | 2 | 34°50´18´´ | 103°10´33´´ | 3055 | Potentilla fruticosa, *Carex capillifolia*, *Medicago ruthenica* |
|  | 3 | 34°50´33´´ | 103°01´25´´ | 3208 | *Carex capillifolia*, Potentilla fruticosa, *Potentilla fragarioides* |

**Table S2.** The effects of pollination treatment (open pollination vs. supplemental pollination) and distance to oilseed rape fields (near vs. far) on plant traits and seed set analyzed by two-way ANOVA. Bold indicate statistically significant effects.

| Co-flowering communities | Trait | Distance | |  | Pollination treatment | |  | Distance × Pollination treatment | |
| --- | --- | --- | --- | --- | --- | --- | --- | --- | --- |
|  |  | *F_1,284_* | *p* |  | *F_1,284_* | *p* |  | *F_1,284_* | *p* |
| Alpine meadow | Flower height | **52.023** | **<0.001** |  | **8.443** | **0.004** |  | 2.831 | 0.094 |
|  | Flower size | 1.105 | 0.291 |  | 1.180 | 0.123 |  | 0.880 | 0.163 |
|  | Seed set | **4.320** | **0.016** |  | **87.387** | **<0.001** |  | 0.006 | 0.564 |
|  |  | *F_1,339_* | *P* |  | *F_1,339_* | *p* |  | *F_1,339_* | *p* |
| Shrub meadow | Flower height | **5.992** | **0.015** |  | **10.238** | **0.002** |  | 0.191 | 0.662 |
|  | Flower size | 1.586 | 0.240 |  | **6.013** | **0.017** |  | 0.297 | 0.667 |
|  | Seed set | **5.841** | **0.004** |  | **69.138** | **<0.001** |  | 0.298 | 0.686 |

**Table S3.** Linear variance-standardized phenotypic selection gradients (β_i_ ± SE) for flower height and flower size of *T. ranunculoides* in each pollination treatment (Control, C vs. Hand-pollination, HP) and distance to oilseed rape (OSR, Near vs. Far) in alpine meadow and shrub meadow. Statistically significant or marginally significant (0.05 < *p* < 0.1) estimates are in bold. The strength of pollinator-mediated selection (Δβ_poll_ ± SE) and associated *p*-values are also given.

| Co-flowering communities | Distance to OSR | Trait | C | | HP | | Pollinator-mediated selection | |
| --- | --- | --- | --- | --- | --- | --- | --- | --- |
|  |  |  | β_i_±SE | *p* | β_i_±SE | *p* | Δβ_poll_±SE | *p* |
| Alpine meadow | Near | Flower height | **0.292±0.091** | **0.002** | **0.127±0.066** | **0.056** | **0.165±0.112** | **0.024** |
|  |  | Flower size | **0.191±0.091** | **0.039** | 0.104±0.066 | 0.118 | **0.087±0.112** | **0.057** |
|  | Far | Flower height | **0.354±0.108** | **0.002** | 0.043±0.058 | 0.446 | **0.311±0.123** | **0.010** |
|  |  | Flower size | 0.144±0.108 | 0.191 | **0.170±0.058** | **0.005** | -0.026±0.123 | 0.397 |
| Shrub meadow | Near | Flower height | **0.285±0.091** | **0.002** | **0.134±0.064** | **0.041** | 0.151±0.111 | 0.187 |
|  |  | Flower size | 0.074±0.091 | 0.419 | 0.088±0.064 | 0.177 | -0.014±0.111 | 0.944 |
|  | Far | Flower height | **0.249±0.079** | **0.001** | **0.130±0.058** | **0.028** | 0.119±0.095 | 0.250 |
|  |  | Flower size | **0.171±0.075** | **0.024** | **0.192±0.058** | **0.001** | -0.021±0.095 | 0.793 |

**Table S4** Parameter estimates (±SE) from the full models evaluating the effect of plant traits, distance to oilseed rape, and pollination treatment on relative female fitness. Fixed factors included were pollination treatment (Control, C vs. Hand-pollination, HP) and distance (Near vs. Far). Plant traits (Flower height and Flower size) were included as continuous variables. Bold numbers indicate statistically significant estimates.

|  |  |  | Estimate±SE | *p* |
| --- | --- | --- | --- | --- |
| Alpine meadow | (Intercept) |  | **0.965±0.073** | **<0.001** |
|  | Distance |  | 0.055±0.076 | 0.473 |
|  | Pollination treatment |  | 0.065±0.073 | 0.380 |
|  | Flower height |  | **0.354±0.099** | **<0.001** |
|  | Flower size |  | 0.144±0.099 | 0.149 |
|  | Distance × flower height |  | -0.062±0.126 | 0.621 |
|  | Pollination treatment×flower height |  | **-0.312±0.139** | **0.025** |
|  | Distance × flower size |  | 0.048±0.126 | 0.705 |
|  | Pollination treatment×flower size |  | 0.027±0.139 | 0.808 |
|  | Distance × pollination treatment×flower height |  | 0.147±0.173 | 0.396 |
|  | Distance × pollination treatment×flower size |  | -0.115±0.173 | 0.508 |
| Shrub meadow | (Intercept) |  | **1.000±0.057** | **<0.001** |
|  | Distance |  | -0.000±0.066 | 1.000 |
|  | Pollination treatment |  | 0.000±0.066 | 1.000 |
|  | Flower height |  | **0.249±0.072** | **<0.001** |
|  | Flower size |  | **0.171±0.072** | **0.018** |
|  | Distance × flower height |  | 0.037±0.102 | 0.718 |
|  | Pollination treatment×flower height |  | -0.118±0.102 | 0.246 |
|  | Distance × flower size |  | -0.097±0.102 | 0.342 |
|  | Pollination treatment×flower size |  | 0.021±0.102 | 0.838 |
|  | Distance × pollination treatment×flower height |  | -0.034±0.144 | 0.815 |
|  | Distance × pollination treatment×flower size |  | -0.007±0.144 | 0.962 |


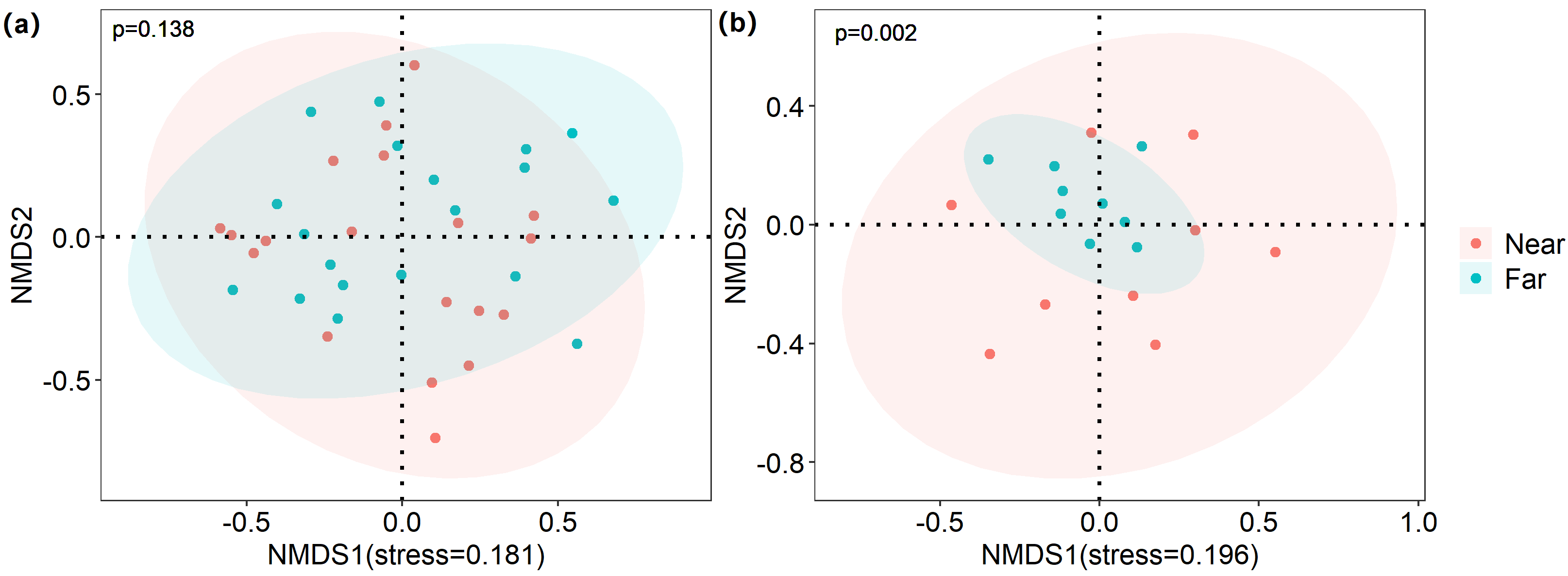


**Figure S1.** Differences of pollinator composition based on Bray-Curtis distances in alpine meadow (a) and shrub meadow (b) with different distances to oilseed rape fields (Near vs. Far).
